# Biology: The Open Road to a Theory of Life

**DOI:** 10.3390/biology13121025

**Published:** 2024-12-07

**Authors:** Andrés Moya, Jukka Finne

**Affiliations:** 1 Integrative Systems Biology Institute (I2Sysbio), University of Valencia and Spanish Research Council (CSIC), 46980 Valencia, Spain; 2 Foundation for the Promotion of Sanitary and Biomedical Research of Valencia Region (FISABIO), 46021 Valencia, Spain; 3 Biomedical Research Network Center in Epidemiology and Public Health (CIBEResp), 28029 Madrid, Spain; 4 Molecular and Integrative Biosciences Research Programme, Faculty of Biological and Environmental Sciences, University of Helsinki, PL 56, FI-00014 Helsinki, Finland

## 1. Biology: The Great Science

The journal *Biology* was launched in 2012. Its first publication was an Editorial by its first editor, Prof. Christopher A. O’Callaghan [1], entitled “Biology: the path ahead”. But that road ahead is still a wide open one, with many possible new routes. Nothing could be more appropriate to define this science than to consider the long road ahead. Life, in all its vastness, is the generic objective of the study of Biology. This study is carried out using two fundamental approaches. The first one consists of investigating the organisms themselves, as a whole, and their classification and systematics. The second approach considers their scales of organization, from molecules to ecosystems, passing through cells, organs, tissues, individuals, populations, and species.

If we consider the types of organisms, we have the classical disciplines that study life, such as Microbiology, Zoology, or Botany. However, let us look at the scales of organization. There are transversal disciplines that, by dissecting organisms or considering their groupings, delve into their structure, organization, and function at these scales. Thus, we can include Molecular Biology, Genetics, Biochemistry, Cell Biology, Evolution, and Ecology. Both in terms of the types of organisms and the scales of organization, the different biological disciplines seek to explain observations, phenomena, or regularities, as well as discovering laws or theories that are specific to each organism or level. Without delving into the classic debate on whether the theories on biological typology or specific scales of organization are reducible to the theories of others, the fact is that Biology as a science is in the process of being able to carry out an integrative treatment of the observations and results of biological types and their levels of organization. This type of study has a certain novelty with respect to the two fundamental types of research mentioned above, which we could call “Integrative and Systems Biology”. We are not defining a new discipline of Biology; instead, we are indicating that the accumulation of information coming from the classical sciences of Biology and its integration is allowing us to enter the field of deeper explanations of life, its evolution, and, to a certain extent, its recreation, all of which are well supported empirically.

As is the case with any science, the road ahead of Biology as a scientific field is endless. We cannot say that science ends with the discovery of final theories that can be considered definitive. That is the case with Biology, too. The consolidation of Biology as a science, contrary to what it may seem, is relatively recent and can be traced back to the moment when Darwin formulated his theory of evolution by natural selection. Of course, there is a great deal of research on living beings that preceded Darwin’s. Still, it is not easy to find a general theory of living beings before him because religious, philosophical, or ideological presuppositions have always conditioned the nature of living features with little scientific basis and went beyond or had little to do with biological science. A metaphor that appropriately illustrates this circumstance is the fact that rather than “reading from the book of Nature, biological features were read or interpreted from other sources”. These assumptions have greatly conditioned the consolidation of Biology and delayed the discovery of laws, theories, or regularities that are so frequent within other sciences, such as Physics or Chemistry. Notwithstanding this circumstance, it is also true that the very complexity of the phenomenon of life, its origin, evolution, and multilevel organization contribute to the slow discovery of new laws and theories.

An essential unit that encapsulates such integration is the cell. Let us consider the cell as the fundamental unit of life. We can indicate that current and future Biology pursues the formulation of a theory of “cellular life” that is capable of explaining its origin, development, and evolution. However, this formulation is not a speculation or generic reflection, as it was in the past when Biology as a science began to take shape. We now have much more solid empirical and experimental support that comes from the integration of all available information on organisms and their levels of organization [2,3]—and we will have much more in the future.

## *2. Biology*: The Journal

*Biology* intends in this second period to develop an Editorial line under the criterion of advancing Biology as a science in the discovery of regularities, laws, and theories within the different levels of organization of life, as well as between levels, under this fundamental consideration of progress towards a theory of the cell as a basic theoretical framework. The central point is that the studies published should contribute to the theoretical advancement of Biology and the finding of generalities or regularities within and between the different levels of organization of life. It will be important to take into consideration not only new experimental and observational advances with the implementation of new methods of the different sciences but also, very importantly, the reinforcement with biocomputing, modeling, and theoretical developments in complex systems.

Although Biology embraces a wide variety of fields, a common denominator in *Biology* is the study of life. We do not make a distinction between different fields as long as the contributions add to the common goal. Many fields within or overlapping to various degrees with Biology have their own identity and tradition, including their well-established publication channels. Contributions from these fields are welcome as long as they significantly contribute to the general scheme of the study of life. However, studies dealing with highly specific or practical questions that are inherent to a particular field should preferably be directed to specialty journals, where the contributions are more likely to find their appropriate readership.

## 3. Publish in *Biology*

It is nothing new to say that peer review is critical in science. However, we want to add that the most appropriate and qualified experts should carry out this review. The journal has incorporated in its Editorial Board a very high number of expert and well-qualified scientists working in Biology who fall within the defined areas in which we have distributed the fields of biological knowledge (https://www.mdpi.com/journal/biology/about, accessed on 3 December 2024).

It is an exciting opportunity to submit your articles to *Biology* now.
